# Anastomotic Strictures after Esophageal Atresia Repair: Incidence, Investigations, and Management, Including Treatment of Refractory and Recurrent Strictures

**DOI:** 10.3389/fped.2017.00120

**Published:** 2017-05-29

**Authors:** Renato Tambucci, Giulia Angelino, Paola De Angelis, Filippo Torroni, Tamara Caldaro, Valerio Balassone, Anna Chiara Contini, Erminia Romeo, Francesca Rea, Simona Faraci, Giovanni Federici di Abriola, Luigi Dall’Oglio

**Affiliations:** ^1^Digestive Endoscopy and Surgery Unit, Bambino Gesù Children’s Hospital, IRCCS, Rome, Italy; ^2^University of L’Aquila, L’Aquila, Italy

**Keywords:** esophageal atresia, anastomotic strictures, esophageal dilation, bougie dilators, balloon dilators, refractory and recurrent strictures, adjuvant treatments, esophageal stenting

## Abstract

Improved surgical techniques, as well as preoperative and postoperative care, have dramatically changed survival of children with esophageal atresia (EA) over the last decades. Nowadays, we are increasingly seeing EA patients experiencing significant short- and long-term gastrointestinal morbidities. Anastomotic stricture (AS) is the most common complication following operative repair. An esophageal stricture is defined as an intrinsic luminal narrowing in a clinically symptomatic patient, but no symptoms are sensitive or specific enough to diagnose an AS. This review aims to provide a comprehensive view of AS in EA children. Given the lack of evidence-based data, we critically analyzed significant studies on children and adults, including comments on benign strictures with other etiologies. Despite there is no consensus about the goal of the luminal diameter based on the patient’s age, esophageal contrast study, and/or endoscopy are recommended to assess the degree of the narrowing. A high variability in incidence of ASs is reported in literature, depending on different definitions of AS and on a great number of pre-, intra-, and postoperative risk factor influencing the anastomosis outcome. The presence of a long gap between the two esophageal ends, with consequent anastomotic tension, is determinant for stricture formation and its response to treatment. The cornerstone of treatment is endoscopic dilation, whose primary aims are to achieve symptom relief, allow age-appropriate capacity for oral feeding, and reduce the risk of pulmonary aspiration. No clear advantage of either balloon or bougie dilator has been demonstrated; therefore, the choice is based on operator experience and comfort with the equipment. Retrospective evidences suggest that selective dilatations (performed only in symptomatic patients) results in significantly less number of dilatation sessions than routine dilations (performed to prevent symptoms) with equal long-term outcomes. The response to dilation treatment is variable, and some patients may experience recurrent and refractory ASs. Adjunctive treatments have been used, including local injection of steroids, topical application of mitomycin C, and esophageal stenting, but long-term studies are needed to prove their efficacy and safety. Stricture resection or esophageal replacement with an interposition graft remains options for AS refractory to conservative treatments.

## Introduction

Since the original description of successful repair and primary anastomosis in 1943 (1) improved intensive care treatment, anesthetic techniques, and surgical techniques have dramatically raised survival rates in esophageal atresia (EA). Therefore, the long-term morbidity in children, adolescents, and adult EA patients has become a common challenge for clinicians (2). Anastomotic strictures (ASs) are still the main complication after repair of EA in neonates (3, 4). Despite the identification of multiple risk factors for ASs, such as long-gap EA with consequent anastomotic tension, postoperative anastomotic leak, and gastroesophageal reflux disease (GERD) (3, 5–11), prevention strategies with intraoperative techniques and/or postoperative treatments have failed to decrease the incidence of ASs over time (12). Traction and growth surgical techniques are considered a good system to induce esophageal growth and elongation, therefore facilitating anastomosis with less tension (13). The possible role of these procedures in preventing AS formation has to be clarified. Currently, the burden of ASs in the postoperative care of AE patients is still high and requires improvement of treatment strategies, especially for refractory and recurrent strictures.

The European Society for Pediatric Gastroenterology Hepatology and Nutrition (ESPGHAN) and the North American Society for Pediatric Gastroenterology, Hepatology and Nutrition (NASPGHAN) recently published the first Guidelines for the evaluation and treatment of gastrointestinal and nutritional complications in children with EA, including indications for diagnosis and management of ASs (14). Nonetheless, prospective studies are still required to optimize strategies to prevent, investigate, and effectively manage patients with ASs after EA repair (12). This review evaluates the recent literature surrounding ASs, with particular focus on refractory and recurrent strictures. We analyzed and compared selected studies, based on existing theories, models, and experts’ opinion. Given the lack of evidence-based data on AS in EA children, in order to provide a comprehensive view of the topic, we also critically discussed results coming from adult literature or from studies on esophageal strictures caused by different etiologies.

## Definition and Diagnosis

Anastomotic stricture is defined as a narrowing at the level of the esophageal anastomosis, detected by barium contrast study and/or endoscopy, and associated with significant functional impairment and symptoms (14). Gastrointestinal symptoms include feeding and swallowing difficulties, drooling, regurgitation and vomiting, foreign body impaction, and poor weight gain. Respiratory symptoms include cough, oxygen desaturation during feeding, aspiration, and recurrent respiratory infections (14).

Diagnostic techniques include esophageal contrast X-ray and endoscopy, with different advantages of the two techniques. Radiological images show the esophageal morphology and may detect associated anomalies (i.e., congenital esophageal stenosis) and pulmonary problems, while endoscopy allows combined diagnosis and treatment (14). Measurements are easier on the static radiologic images, while the endoscopic view may induce errors due to prospective effects; nonetheless, they can be minimized by shooting an instant view when endoscope lens is at a given distance (i.e., 2 cm) proximal to the identified AS (15). Simultaneous visualization of a probe with known diameter (guide wire or plastic tube) may help in measuring the degree of luminal narrowing.

There is no consensus about the fluoroscopy or endoscopy definitions for AS in pediatrics. The reduction of luminal diameter must be compared to an age-related normal esophagus (16). Said et al. proposed the stricture index, SI = *D*−*d*/*D*, where *D* is the esophageal diameter of lower pouch and *d* is the stricture diameter (17). Although the SI has already been used in some studies to assess the degree of ASs by radiographic (18) and endoscopic measurements (15), its clinical usefulness and impact must be confirmed in larger series. More recently, Sun et al. proposed the Esophageal Anastomotic Stricture Index (EASI), as a predictor of the development and severity of ASs after EA repair (19). The EASI was generated after fluoroscopic evaluation of the upper gastrointestinal tract in the early postoperative period (postoperative days 5–10). The equation is a ratio between the diameter of the stricture and the diameter of the upper (U-EASI) and lower (L-EASI) pouches: EASI = (lateral *d*/*D* + anteroposterior *d*/*D*)/2, where *D* is the esophageal diameter of upper or lower pouch and *d* is the stricture diameter. The average between anteroposterior and lateral diameters is considered. The result expresses the diameter of the anastomosis as a percentage of the diameter of the patient’s normal esophagus. The authors conclude that EASI is a simple, reproducible tool to identify patients at risk of ASs, to guide the frequency of follow-up visits as well as the scheduling of contrast studies or upper endoscopy, to correlate the severity of strictures with the efficacy of various treatment methods, and to compare anastomotic techniques in patient registries (19). Further studies are needed to validate its usefulness and reproducibility.

The timing for the first screening or assessment of suspected AS is not universally accepted. It is true that most surgeons perform a baseline barium swallow about 5–10 days postoperatively to rule out the presence of an anastomotic leak, thus giving an early postoperative picture of esophageal lumen (19). Nevertheless, a varying degree of “physiological” stenosis can be found on the first esophagram as a normal healing process from the surgical procedure, without relation to the development of a clinically relevant stricture (15). Early routine screening for ASs, starting 1 month after surgery, has been proposed (15). Recent guidelines, according with the majority of authors, recommend that AS should be excluded only in symptomatic children and those who are unable to achieve feeding milestones (14, 20).

Concerning clinical signs and symptoms, it is worth to underline that in EA children gastrointestinal and respiratory manifestations secondary to AS may overlap with other pathologic conditions, such as esophageal dysmotility, recurrent tracheoesophageal fistula, GERD, tracheomalacia, laryngeal clefts, and vocal cord dysfunction (12). Clinicians must be aware that these conditions may coexist and exacerbate AS symptoms. Moreover, the degree of esophageal narrowing does not correlate with symptoms. Therefore, patients with EA should be evaluated regularly by a multidisciplinary team to rule out the presence of other comorbidities (14).

Once an AS has established, the cornerstone of treatment is endoscopic dilation, whose primary aim is to achieve symptom relief (14). Unfortunately, some patients may experience symptoms persistency or recurrence despite multiple dilation sessions. A definition for both refractory and recurrent strictures has been proposed by Kochman et al. for adults, based on lumen diameter: refractory stricture refers to the inability to successfully remediate the anatomic problem to a diameter of 14 mm over five sessions at 2-week intervals, while recurrent stricture is the inability to maintain a satisfactory luminal diameter for 4 weeks once the target diameter of 14 mm has been achieved (21). A definition for pediatric patients has been proposed using the SI, with refractory stenosis defined as the persistence of SI > 10% after five dilation sessions, and recurrent stenosis in case of recurrence of symptoms or SI > 50% once that SI < 10% has been achieved (12). Expert opinion by the working group of ESPGHAN–NASPGHAN Guidelines for EA patients is that recurrent AS may be defined as ≥3 episodes of clinically relevant stricture (14). More recently, based on Kochman criteria, the ESPGHAN–ESGE Guidelines on diagnostic and therapeutic endoscopy in pediatrics suggests the following definitions: “inability to successfully remediate the anatomic problem to obtain age-appropriate feeding possibilities after a maximum of 5 dilation sessions with maximal 4-week intervals” for refractory stricture; “inability to maintain a satisfactory luminal diameter for 4 weeks once the age-appropriate feeding diameter has been achieved” for recurrent stricture (20).

Refractory and recurrent ASs are a major challenge in the postoperative management of AE patients, and a better understanding of risk factors is essential for prevention strategies, as well as the improvement of therapeutic approaches.

## Incidence and Risk Factors

The normal process of wound healing after creation of the esophageal anastomosis involves tissue remodeling and wound contraction, promoted by fibroblasts. Wound contraction in the setting of a circular end-to-end anastomosis creates narrowing. Therefore, it is quite natural to see a degree of narrowing at the site of the esophageal anastomosis after EA repair (22).

Reported incidence of AS after AE repair ranges from 32 to 59% in the majority of recent studies (3, 4, 7, 9, 23–25), but also lower and higher incidence has been reported, up to 5% (26) and 80% (27), respectively. This variability relies in different definitions of AS and in a great number of pre-, intra-, and postoperative risk factor influencing the anastomosis outcome. These risk factors may affect stricture formation as well as its response to treatment, leading to recurrent and refractory ASs.

### Preoperative Risk Factors

Gestational age, AE type and associated malformations, and length of the gap have been proposed as preoperative risk factors.

A relationship between stricture formation and prematurity or low birth weight, as well as VACTERL association, has been reported in retrospective cohorts (7, 8), but not confirmed in other series (5). The stricture rate seems to be unaffected by sex and intrauterine growth retardation (7). The role of tracheomalacia is controversial, since some authors reported its association with anastomotic complications (5), while others did not (7).

The type of EA may affect the incidence of AS, due to the different length of upper and lower esophagus, which are manipulated and mobilized during surgery (28). Indeed, vascular compromise affects especially the lower esophagus, which has a segmental blood supply from the aorta or the intercostal blood vessels. Mobilization of the lower esophagus may risk devascularization, ischemia at the esophageal ends, and stricture formation. Conversely, mobilization of the upper esophagus can be performed without vascular compromise, as this segment has a good blood supply coming from the inferior thyroid artery (28).

Long-gap EA is considered a significant predictive factor for developing early and late ASs, defined as strictures occurring less or more than 1 year after surgery, respectively (3). However, it is worth to point out that there is no univocal definition of long gap. It can be measured in centimeters or vertebral bodies. Some authors define 2 or 3 or 3.5 cm as a cutoff point; others classify the gap into short (1 cm), intermediate (2.5–3 cm), and long (>3 cm); others recommend an esophageal replacement if the gap exceeds the length of six vertebral bodies (13).

### Intraoperative Risk Factors

Intraoperative risk factors for ASs include tension of the anastomosis, degree of ischemia, and type of suture.

The surgical attitude toward AE repair has changed over the last decades, with an increased rate of early primary esophageal repair and a respective reduction of delayed primary repair and esophageal replacement (29). This changing may be attributed to several factors: improved neonatal care allows children to be in a better condition to survive early definitive surgery; moreover, increased understanding and specialized training of neonatal surgeons has made primary esophageal reconstruction achievable in most long-gap patients (13). A direct consequence of primary anastomosis in long-gap AE is anastomotic tension, which in turn contributes to AS, as widely reported in retrospective analysis (5–11). Nonetheless, with meticulous handling of the esophageal ends, preservation of the blood supply, and care to include the mucosa in each and every suture of the anastomosis, strictures can be kept to a minimum (24). Surgical techniques of esophageal lengthening, which have been used to achieve a primary anastomosis, may facilitate the reduction of anastomotic tension (13). The Kimura advancement technique may be applied only to the upper esophageal pouch and consists on multistage extra thoracic esophageal elongation of the proximal esophagus by moving the cutaneous stoma progressively further down the anterior chest wall (13, 30). Foker technique involves extensive mobilization of both upper and lower pouches and the placement of sutures in both segments, which are brought out to the skin surface and progressively pulled in the following days until a primary repair is possible (13, 30–32). Further prospective studies are required to investigate the possible protective role of these techniques against AS development.

The type of suture may influence AS formation (28): ASs have been reported to be less frequent when absorbable sutures are used for the initial repair, although this was not confirmed in experimental studies; interrupted sutures are used to potentially reduce the risk of stricture; two-layered or the Haight anastomosis and end-to-side anastomosis are associated with an increased incidence of stricture.

Incidence of ASs seems not to be influenced by thoracoscopic approach versus thoracotomy (33–36).

### Postoperative Risk Factors

Anastomotic strictures’ formation is influenced by postoperative risk factors, including anastomotic leak and GER. Anastomotic leakage is reported to be more frequent in long-gap AE (8) and to predispose to AS (10, 11). The role of GERD has been diffusely investigated. Mobilization of the distal esophagus and superior displacement of the esophagogastric junction promote GERD (9) and the exposure of the anastomotic area to acid secretions may enhance the reparative response and facilitate stricture formation as well as recurrence or resistance to treatment. Retrospective series reported an increased incidence of ASs in children with GERD (3, 4). AS is reported as a possible complication also in the 8–15% of adult AE patients; as these strictures most likely arise as a result of prolonged acidic reflux, the far-reaching significance of GERD in these adult patients is further underlined (37). However, a multivariate analysis showed the absence of an association between GERD and subsequent stricture formation, probably due to the prescription of a systematic proton pump inhibitor (PPI) (7). An extensive description of GERD diagnosis and management has been reported in the cited ESPGHAN–NASPGHAN Guidelines, including medical and surgical treatment (14). Even if evidence of the beneficial role of prophylactic PPI therapy is lacking in retrospective studies (38), the panels suggest a systematic routine treatment with PPI for 1 year after surgical correction, also in asymptomatic patients. It will be interesting to investigate whether this routine practice will decrease the stricture formation in the future (9).

Duration of intubations after AE repair was associated with increased risk of AS in some patients (8), but not in other cases (7).

## Treatment

Management of ASs implies a multistep and multidisciplinary approach. Endoscopic dilations are the mainstream of the conservative approach and may benefit from other adjuvant strategies for refractory and recurrent ASs (14, 20). Treatment of comorbidities is essential for the global care of each patient. Surgical approach must be reserved to extremely selected patients (39, 40).

### Esophageal Dilation

By exerting expansible forces within the lumen of the stenosis, dilations result in an increased esophageal diameter. Since the first pediatric description approximately 30 years ago (41), esophageal dilation has become the recommended first-line treatment for AS following EA repair (14).

The primary goal of esophageal dilation is to achieve symptom relief, permit maintenance of age-appropriate oral nutrition, and reduce the risk of pulmonary aspiration.

Two main categories of dilators are used in gastrointestinal endoscopy: fixed-diameter push-type dilators (bougie dilators) and radial expanding balloon dilators (42).

#### Bougie Dilators

Several bougies, varying on designs, calibers, and lengths, are available, but they may be further subdivided into two main categories: weighted (tungsten-filled) or wire-guided bougies.

Flexible tungsten-filled bougies do not accommodate a guidewire and are generally passed blindly without fluoroscopic assistance. Patients may be instructed to use for self-dilation at home. Hurst (Milwaukee, WI, USA) and Maloney dilators (Milwaukee, WI, USA) are the most commonly used non-wire-guided bougies and differ by their tips. The former has a rounded blunt tip, whereas Maloney dilators have an elongated tapered tip. The blind passage of non-wire-guided bougies may lead to a higher risk of perforation and to the incorrect passage of a dilator into the trachea (42, 43).

Wire-guided dilation provides assurance that the dilator is following the line of the esophageal lumen, so they are generally preferred (44).

The most popular guidewire-assisted mechanical bougies are the polyvinyl Savary–Gilliard dilators (Cook Medical, Bloomington, MN, USA). They have a long-tapered tip and a radiopaque band at the beginning of the widest portion of the dilator to allow fluoroscopic guidance. After the tip of the guidewire is endoscopically placed across the stricture, the endoscope is withdrawn, and dilator is passed over the wire. All steps may be monitored with fluoroscopic aid, especially if the endoscope does not traverse the stricture (42).

Fixed-diameter bougie dilators exert radial forces and also cause a shearing effect that generates longitudinal forces as they are passed across the stenosis. The dilation is achieved by using gradually increasing dilator diameters. The selection of the initial size of dilator is based on an estimation of the stenosis diameter. Dilation is considered to have been performed when there is a moderate or significant amount of resistance. Contrarily to balloon dilation, bougie dilation is a tactile technique, meaning that the operator may feel the amount of resistance encountered with passage through the esophagus and apply the correct force to overcome the stenotic area.

Although there are no definite evidences, it is generally accepted that the risk of perforation could be minimized if the “rule of three” is applied, meaning that, after moderate resistance is encountered, no more than three dilators of progressively increasing diameter should be passed in a single session (20, 45). However, especially in pediatrics, operator experience plays a pivotal role in the choice of the optimal dilator size. Endoscopic assessment of the tissue damage is advised after each dilation, to guide decision-making (40). Bougies are more cost-effective than balloon dilators because they are reusable.

#### Balloon Dilators

Balloon dilators only exert radial forces when expanded within a stenosis. In contrast to what occurs with bougies, if the balloon is longer than the stricture, the force is delivered simultaneously over the entire length of the stenotic segment rather than progressively from its proximal to its distal extent (42).

Balloon dilators designed for single use only are available in an array of designs, lengths, and calibers. Dilations can be performed under endoscopic guidance with or without fluoroscopy in the operating room or under fluoroscopic guidance in the radiology suite. Through-the-scope (TTS) balloon dilators (Boston Scientific, Marlborough, MA, USA) are currently by far the most frequently used. They are designed to pass with or without guidewire. TTS balloon dilators are passed through the endoscope working channel, which enables the procedure to be performed under direct vision. The balloon is placed across the stenosis and expanded with water or contrast by using a handheld inflation device. The hydraulic pressure can be monitored manometrically. Newer TTS are designed to produce three distinct diameters at three separate pressures during *in vivo* dilation. Despite there are no data on the optimal time the balloon should remain inflated, in practice the inflation pressure is maintained for approximately 30–60 s or until there is a sudden drop in manometric pressure. If fluoroscopy is used, successful dilation is detected by the obliteration of the “waist” on the balloon, representing the stricture. A drawback for TTS balloons is that they require a 2.8-mm working channel and then they are not compatible with small-caliber pediatric endoscopes. In younger children, the balloon can be positioned over a guidewire under fluoroscopic guidance.

The serial incremental size of TTS balloon dilators per single session can follow the “rule of 3,” as described earlier for bougie dilators (20).

#### Bougie versus Balloon Dilators: Outcome and Comparative Data

Despite advances in endoscopic equipment and dilators have improved the safety of esophageal dilation, the procedure may lead to complications even in the most experienced hands. The most frequently reported complications of esophageal dilation include perforation, hemorrhage, and bacteremia. In adults, the overall perforation rates vary between 0.1 and 0.4% (44, 46).

Long-term outcomes are influenced by the underlying condition; stricture diameter and length are established factors that influence the number of dilations required for symptom relief and the need for additional dilations (47). Children with long-gap EA and postoperative anastomotic leak are more prone to develop more severe AS.

A systematic review, including 5 studies (17, 48–51), has looked at the outcomes of balloon dilation (fluoroscopic and/or endoscopic) in children with EA (139 children with a total of 401 balloon dilation sessions), reporting a success rate ranging from 70 to 100%, with approximately 3 dilation sessions per child and a perforation rate of 1.8% (52). Alshammari et al. analyzed a series of 49 children who underwent esophageal balloon dilation for different etiologies; among 24 EA children they reported a median of 2 dilatations per patient, with a perforation rate of 8% (2 patients) (53). In a study aimed to retrospectively evaluate efficacy and complications of esophageal dilatations with Savary–Gilliard bougies in 23 children with EA, dilation was successful in 87% of patients, stricture resolution occurred after a mean of 3.2 dilatations per patient, and no complications were observed during or after the dilatation sessions (7). Moreover, in a large study, 107 children with benign esophageal strictures underwent Savary–Gilliard bougie dilations, the procedure was successful in 104 patients (93.7%), and perforations occurred in 6 cases during 648 dilation sessions (0.9%). In this study, only 12 children had AS secondary to EA, while most patients had caustic strictures (54).

Two retrospective studies involving EA children compared the two techniques. Lang et al. reported that children with EA who had undergone balloon dilation (16 patients) required fewer procedures than the bougie group (12 patients) (2.0 versus 8.5, respectively), while perforations (2 cases) occurred only after balloon dilation (52). Jayakrishnan and Wilkinson reported that fluoroscopic balloon dilatation (125 procedures) had fewer perforations than surgical bouginage (88 procedures) (1.6 versus 5.7%, respectively) in 37 children with esophageal strictures (24 with EA) (55).

Currently, there are no randomized controlled trials comparing efficacy and safety of hydrostatic balloon with bougie dilator for treatment of AS in EA children. Data coming from controlled trials in adults found no significant differences between bougie and balloon dilators in terms of efficacy and safety while treating benign esophageal strictures (46, 56).

Therefore, due to the lack of strong evidences, the choice between balloon dilation and bougie is only based on the endoscopist’s experience and level of comfort. Indeed, more than the technique itself, a trained operator is required to reduce complications following esophageal dilations. Based on experts’ opinion, ESPGHAN–NASPGHAN Guidelines for children with EA only recommend the use of guide wire-guided dilators (bougie or balloon) (14).

#### Timing of Dilations: Prophylactic versus Selective Dilatations

Definitely, the degree and duration of the effect of dilation, as well as and the need for repeating the procedure, are dependent on the length and diameter of stenosis, which are in turn linked to the baseline and underlying condition, such as long-gap AE and the presence of severe GERD.

However, there is currently no definitive evidence to support the ideal interval between the dilatation sessions. Based on single institutional experience, various “philosophies” have been adopted in clinical practice, but two main approaches exist: (1) prophylactic routine dilation/calibration to prevent symptoms developing (51) and (2) selective dilatations only when the symptoms arise (7). The rationale of the first approach, performing dilations systematically even in the absence of symptoms, is to ascertain an adequate caliber of the esophagus in all patients and thus avoid complicated strictures and long-term functional problems. The purpose of the second approach, “wait and see,” is to reduce the number of invasive procedures and thus the risk of dilation-related complications. In 2009, Koivusalo et al. retrospectively compared the effect of the two approaches and concluded that the policy of selective dilatations resulted in significantly less dilatations than routine dilation with equal long-term outcomes in terms of dysphagia, nutritional status, and respiratory symptoms (57).

Recent ESPGHAN–NASPGHAN recommendations state that there is no evidence supporting the use of the more invasive strategy of routine dilations; therefore, the presence of AS should be excluded and treated only in symptomatic children (14). However, a close follow-up should be undertaken during the first 2 years of life, with special attention to weaning phase. Patients with long-gap EA and postoperative anastomotic leak need a close follow-up to avoid development of a severe AS.

### Refractory and Recurrent Strictures: Adjuvant Treatments

Despite dilation treatment, some patients may experience symptoms relapse or persistency. The cause of recurrent and refractory AS is not fully understood. As previously discussed, numerous baseline conditions, as well as intra- and postoperative risk factors, concur to the stricture outcome. The dilation procedure itself may be partially responsible, because of intense fibrogenesis during healing process after the dilation procedure. Iterative dilations increase the risk of complications and may cause psychological problems in children. Nevertheless, once a stricture becomes refractory to esophageal dilation, conservative approach is preferable before the patient is candidate to surgery (39). Despite the absence of specific controlled trials, different non-surgical adjuvant treatments can be used in clinical practice for refractory and recurrent esophageal AS.

#### Intralesional Steroid Injection

Intralesional corticosteroid injection as an adjunct to dilatation has been proposed to prevent stricture recurrence approximately 50 years ago (58). However, in the last two decades, there has been a growing interest in the use of this therapy for refractory benign esophageal strictures of various etiologies (59).

Despite this long experience, the real mechanism of action of this treatment remains poorly understood. It is believed that steroid injection may reduce collagen synthesis, fibrosis, and chronic scarring processes, by inhibiting the transcription of certain matrix protein genes (59).

The most used steroid for intralesional injection is triamcinolone acetate or acetonide; betamethasone and dexamethasone preparations have been also used (59).

Triamcinolone acetate (dose 10 or 40 mg/mL; volume per injection ranging from 0.5 to 2.8 mL) is usually injected with a standard sclerotherapy needle in four quadrants of the esophagus at the upper border of the stricture before dilatation (59–61).

Two small-sized randomized trials in adult with recalcitrant esophageal stricture showed that local steroid injections resulted in a decreased need for multiple dilations and a longer average time to repeat dilation. In these series, all but 4 patients (from the Altintas’ study) who underwent steroid injection of the stricture had peptic injury (25 patients in total) (61, 62). The efficacy of steroid injection as adjunctive treatment remains unclear in other types of benign strictures (63). Apart from encouraging results reported in uncontrolled studies (59), well-designed studies showed mixed findings. Hirdes et al., in a multicenter double-blind placebo-controlled trial, failed to find any statistical significance in patients with benign esophagogastric ASs (64). Later, Pereira-Lima et al., in a double-blind randomized trial, reported a significant improvement or resolution of dysphagia with complex esophagogastric anastomotic treatment-naive strictures (65). Camargo et al. found no difference in dilation frequency or recurrent dysphagia in patients with caustic strictures treated by steroid injection or placebo (66). Conversely, Nijhawan et al., treating 11 patients with refractory corrosive esophageal strictures, showed a significantly improved periodic dilation index (number of dilatations per month) and dysphagia score from pre- to postintervention period (67).

Concerning AS in EA children, Gandhi et al. described 12 patients, among which 5 were EA survivor, how received intralesional steroid injections combined with dilations reporting a long-term remission of symptoms (68). Holder et al. and Zein et al. also reported good outcomes in three and one EA children, respectively (69, 70). Even though other centers probably use intralesional steroid injection in clinical practice (15, 22, 71), evidences in EA children are lacking.

Potential complications of esophageal injections of steroid injection include adrenal suppression, perforation, intramural infection, candida infection, mediastinitis, and pleural effusion (71).

Concluding, since studies exploring efficacy and safety of intralesional steroid treatment are small, uncontrolled, and heterogeneous, it is difficult to draw definitive conclusions regarding the benefit of intralesional steroids in reducing recurrent stricture formation in EA patients (14, 71). The ESGE–ESPGHAN Guidelines for pediatric gastrointestinal endoscopy do not support the routine use of intralesional steroids for refractory esophageal stenosis in children (20).

#### Systemic Steroid Therapy

The use of systemic steroids associated with endoscopic dilation has been reported only in anecdotal cases. Hishiki et al. described the case of a boy with EA, who developed refractory AS and underwent surgical resection of the stenotic lesion with reanastomosis. A secondary AS was again impossible to treat with dilations, but ultimately resolved after two short courses of intravenous dexamethasone (1 mg/kg) (72). Morikawa et al. reported the use of high-dose methylprednisolone in a patient with refractory AS who was a candidate for surgical intervention. A scheme with gradual tapering (daily 25, 15, 10, 5, and 2 mg/kg for 4 days each) was administered intravenously after balloon dilation with intralesional steroid injection and followed buy oral prednisolone (daily 2, 1, and 0.5 mg/kg for 1 week each). This treatment finally resolved the AS (73).

Evidence is currently lacking to suggest systemic steroids in AS (14).

#### Mitomycin C (MMC)

Mitomycin C is a natural antitumor antibiotic isolated from the broth of *Streptomyces caespitosus*. MMC can be administered intravenously, to treat upper gastrointestinal cancers (e.g., esophageal and gastric carcinoma), pancreatic adenocarcinoma, and other types of solid cancer. It may also be administered also topically, to treat bladder and intraperitoneal tumors.

In addition to its antineoplastic properties, it has been shown that MMC may inhibit wound healing by downregulating the gene expression for extracellular matrix proteins and then it acts as an antiproliferative agent by decreasing collagen synthesis and scar formation (74). Over the past years, MMC has gained wide acceptance as adjunctive treatment in the field of ophthalmology for reduction of scar formation in glaucoma filtration or pterygium surgery (75). In a study on human Tenon’s capsule tissue, MMC caused almost complete inhibition of fibroblast proliferation. Nonetheless, several factors may influence its efficacy. These factors include the dose delivered to the tissues (which is concentration dependent), volume, duration of exposure, preparation method, administration, and tissue-related factors (76). Following these observations, the use of MMC was extended to the treatment of laryngeal and tracheal stenosis (77) and then esophageal stricture (78).

The delivery method of MMC is an important aspect to be considered; in fact, the application should be targeted precisely to the stenotic segment, while potentially dangerous exposure to the surrounding healthy mucosa should be avoided. Different application techniques have been described, the most frequent was local application *via* a cotton pledget soaked in MMC solution under direct endoscopic visualization (79). Several techniques to protect the mucosa from contact with the pledget have been reported, such as the use of an overtube or a sheath, and frontloading of the pledget in a standard cap used for band variceal ligation. The use of a drug-eluting microporous polytetrafluoroethylene catheter balloon positioned across the stricture under fluoroscopic guidance was also described (80). Spraying onto the stricture is another possible technique (81). A further alternative, previously reported only in adult studies, involves injection of MMC directly into each quadrant of the stenosis after dilation (82, 83).

Mitomycin C was mostly reported to be freshly prepared immediately before the application. A recent systematic review showed that concentrations of MMC ranged from 0.1 to 2 mg/mL (median and mean values of 0.4 and 0.5 mg/mL, respectively). The number of MMC applications varied between 1 and 12 with a mean of 2 and 2.6 in pediatric and adult patients, respectively, although the majority [67 children (79%) and 24 adults (63%)] required only 1 to 2 applications. When MMC was applied more than once, intervals ranged from 1 week to 13 months, with a median of 4 weeks (84). To date, no study compares the effectiveness of different concentrations and dosages of MMC; the concentration of 0.4 mg/mL is the most commonly used and appears to be effective.

Most data on MMC efficacy in treating persistent esophageal stricture are coming from studies involving patients with caustic esophageal injury (79, 84).

El-Asmar et al., in a double-blinded, randomized, placebo-controlled trial involving children with caustic esophageal strictures, showed a significant reduction of the number of dilatation sessions needed to alleviate dysphagia in patients undergone MMC application compared to controls. During the study period, 80% of strictures in the MMC group got completely resolved compared to only 35% in the placebo group (85). Berger et al. systematically reviewed pediatric studies, showing that in 27 of the 31 children published (87.1%) results were either excellent or good. A complete relief of symptoms was achieved in 21 children (67.7%), partial relief in 6 (19.4%), and no benefit in 4 (12.9%). Importantly, no adverse effect was found in any case. Only 7 out of 31 were EA children, and all but 1 had a good outcome (79). However, the results of a more recent retrospective study involving EA children contradicted these promising results. Chapuy et al. compared the outcome of 11 EA children who received topical application of MMC with 10 EA historical controls who underwent 3 or more dilations. The final outcome was similar in the two groups, with the stricture disappearing in the majority of children. Furthermore, the median number of dilations, although not statistically significant, was smaller in the historical cohort than in the MMC group [3.7 (range 3–7) and 5.4 (range 3–11), respectively]. Author concluded that in EA children adjuvant MMC treatment does not confer a real benefit compared with repeated dilations alone (86).

Potential side effects of systemic MMC include bone marrow, pulmonary, and renal toxicity; however, topical application has not been described to cause severe side effects so far. Nevertheless, being MMC a cytostatic agent, there is a hypothetical risk of secondary malignancy. Indeed, given the rapid cell turnover of the gastrointestinal epithelium, the activity of MMC on esophageal mucosa may lead to dysplastic transformation especially with repeated applications (79). To date, only in one case series, a *de novo* gastric metaplasia at the site of stenosis has been revealed in two out of six patients (39). For this reason, great caution should be taken and a long-term endoscopic follow-up program with esophageal biopsies at the site of MMC application is recommended (39, 79).

Concluding, encouraging data about local MMC are mostly derived from caustic refractory strictures. Several questions have no answers yet, and larger prospective studies are needed to better define optimal application technique, dosage, concentration, duration, and number of MMC applications. Despite contrasting reports exist, MMC can be considered as potential adjuvant treatment for the management of recurrent strictures in EA patients, as stated by the ESPGHAN–NASPGHAN Guidelines (14, 20).

#### Incisional Therapy

Endoscopic electrocautery incisional therapy (EIT) has been used as an alternative option for the treatment of Schatzki’s ring and refractory ASs (87). The basic principle of EIT is the disruption of the fibrotic tissue of the stricture to gain satisfactory lumen diameter with a needle-knife electrocautery. Different EIT techniques have been described with or without dilatation, including electrocautery combined with argon plasma coagulation, or endoscopic scissors, but standard needle knives have been applied most often (88).

Standard needle knife is constituted by a diathermy wire that is pushed out from the tube by a handle mechanism. Insulated-tip knife, consisting of a conventional diathermy needle knife with a ceramic tip, which permits cutting only at the side of the knife, seems to be preferable to minimize the risk of perforation (89). EIT technique consists of multiple radial incisions parallel to the longitude of the esophagus at the stricture site followed by endoscopic balloon dilation. All the steps of the procedure are performed under direct visualization (90). A variation of this technique has been developed by Muto et al. who described a radial incision and cutting method in radial incisions are followed by cutting away of the fibrotic tissue between the incisions (91). Lee et al. described a modified method consisting of the use of a transparent hood attached to the scope tip to reduce unintentional injury during incision (92).

Data on safety and efficacy of EIT are primarily derived from reports in adult patients. EIT therapy has shown exciting results in the treatment of both naïve (as a first-line treatment) (92–95) and refractory strictures (90, 91, 96). In a randomized, prospective study, Hordijk et al. demonstrated that EIT and Savary bougienage were equally efficacious as a primary therapy for previously untreated ASs in adults (95). Muto et al. found that EIT resulted in significantly higher patency rates than repeated dilations in the management of refractory AS (after ≥3 dilation sessions) at 6 and 12 months’ follow-up (91). Patients with long-segment (>1 cm) strictures showed a worse outcome in terms of reoccurrence of the stricture and need of repeated treatments to achieve dysphagia-free status (90, 92). Importantly, EIT has shown a good safety profile; no complications occurred in all (90, 92, 94–96) but one of the studies where all 2 out of 54 patients experienced a pinhole perforation who have been treated conservatively (91).

Very limited data on EIT are available in pediatrics. In retrospective series of seven AE children with refractory AS underwent EIT alone (four patients) or in association with esophageal stenting (three patients), sustained symptom improvement was achieved in all patients, in five of them after a single treatment while an additional treatment was needed in two cases. No severe complications were observed (97).

In summary, despite very limited, especially in children, current evidences suggest that EIT could be considered as an alternative treatment in patients AS, particularly in those with a relatively short length stricture.

#### Esophageal Stenting

Esophageal stent placement is the most frequently used method for palliation of dysphagia from esophageal cancer. Over the last years, temporary stent placement has increasingly been used for refractory benign esophageal strictures in adults (98). Despite there is no specifically designed stents for children, this technique has also gained wide acceptance in pediatrics for the management of refractory and recurrent stricture when medical and endoscopic treatments fail (40, 42).

The rationale of esophageal stenting for refractory strictures is that continuous radially oriented pressure over a long period allows the esophagus to maintain luminal patency while simultaneously stretching the stricture. Remodeling of scar tissue may occur while the stent is in place, which can result in persistent luminal patency and reduced risk of recurrent stricture formation.

As for many of the other treatments, even for esophageal stents, experiences primarily derived from the adult literature. Currently, different designs of esophageal stents are commercially available but they can be divided into three main categories: self-expandable metal stents (SEMSs), self-expandable plastic stents (SEPSs), and biodegradable stents (BDSs) (99, 100).

Self-expandable metal stents consist of woven, knitted, or laser-cut metal mesh cylinders that exert self-expansive forces until their maximum fixed diameter is reached. They are composed of nitinol, a nickel, and titanium alloy. To prevent tissue ingrowth through the stent mesh, SEMs can be fully or partially covered by a plastic membrane or silicone (99). Partial or fully covered SEMs are currently recommended for palliation of malignant dysphagia, only fully covered stent designs can safely be removed after a prolonged time of stenting (98).

Self-expandable plastic stents are constituted by a woven polyester skeleton completely covered with a silicone membrane. Radiopaque markers positioned at the middle and ends of the stent to guide the placement under fluoroscopy (99).

Both SEPS and SEMS are generally deployed *via* a delivery device catheter over a guidewire under fluoroscopic guidance. In contrast to most SEMSs, which are sold in a constrained fashion, the SEPS requires mounting onto the delivery catheter just before use (99).

Biodegradable stents are made from a biodegradable polymer that is slowly absorbed so that, contrarily to SEMSs and SEPSs, BDSs does not need to be removed. BDS maintains its integrity and radial distensile force for approximately 6 weeks and disintegrates in 11–12 weeks after deployment (101). Despite all stent types have been used for the treatment of refractory benign esophageal stricture in adults, only the SEPSs received formal approval for this indication in adults (98).

As commercially available esophageal stents are often inappropriate in size for pediatric patients, airway or biliary stents could be used for children. Airway stents are more rigid than traditional esophageal stents, so the risk of complication is increased, but are available in different size (diameter from 8 to 20 mm and lengths from 2 to 8 cm). Biliary stents are more flexible but are available only in small size (caliber of 8 and 10 mm of and lengths of 4, 6, and 8 cm) (22, 102–104).

In order to overcome these limits, a customized stent has been developed. The “dynamic stents” consists of a plastic or silicon tube, customized in different length and diameter according with the stricture size and level, affixed to a nasogastric tube. The main difference with the other expandable stents is that foods, instead of passing within the lumen of the stent, pass between the stent and the esophageal wall allowing for the long-term improvement of esophageal patency. Intraluminal customized stent is passed under fluoroscopic guidance after stricture dilations (105, 106).

No studies have compared different strategies in terms of stenting duration, so no ideal stenting time has been determined yet. Adults guidelines suggests that fully covered SEMSs or SEPSs should remain in place for at least 6–8 weeks and no more than 12 weeks, to maximize success and to minimize the risk of hyperplastic tissue reaction and stent embedment (98). In pediatric series, the range varies from 7 to 133 days but is more typically 4–6 weeks (104). Complications include potentially life-threatening events such as perforation, hemorrhage, and airway compression but also migration (which is the most frequent complication), granulation tissue, gastroesophageal reflux, and aspiration pneumonia (99). More significantly, massive esophageal bleeding has been reported after stent placement as a cause of arterioesophageal fistulae in two cases of EA children (one death). It is important to underlying that, compared with the general population, EA patients have higher incidence of aortic arch and great vessel anomalies, and consequently they may be more prone to develop this catastrophic complication. Thus, cross-sectional imaging is warranted to evaluate the proximity of great vessels (with or without possible aberrancy) to minimize the risk (107).

In adults, the use of removable stents to treat benign esophageal strictures has yielded contrasting results as summarized in a recent systematic review and meta-analysis. The pooled clinical success rate was 40.5% (95% CI 31.5–49.5%) with no significant differences between patients treated with SEPS and SEMS ant those treated with BDS. The overall adverse event rate was 20.6% (95% CI, 15.3–28.1%) with no significant difference between the three types of stents (108).

Overall, pediatric data on stricture resolution are scarce and heterogeneous, reported success rates ranging from 26 to 86% (103, 105, 109, 110). Data on EA patients are even scarcer. Manfredi et al. in 23 EA patients underwent a total of 40 stenting sessions, reported a success rate of 39 and 26% at ≥30 and at ≥90 days after stent removal, respectively. Both SEPS (14 patients) and fully covered SEMS (26 patients) were used. The mean duration of stent placement was 9.7 days (range 2–30 days) (110).

In a series of predominantly small children (median age 1-year-old child), Best et al. reported that esophageal stenting using and airway stent treatment was successful in all patients, five out of seven had long-gap EA (103). Using the customized “dynamic stent,” the group of Bambino Gesù Children’s Hospital all reported an overall success rate of 89% in a series of 79 children, mostly with caustic strictures. Esophageal stenting (≥40 days of duration of stent placement) was successful in 17 out of 21 children with EA (81%). High-dose systemic steroid therapy (dexamethasone 2 mg/kg/day for 3 day) was administered in all children after stent placement (105, 106).

In conclusion, esophageal stenting is a promising tool for the treatment of recurrent and refractory ASs. Advantages include prolonged maintenance of luminal patency and better oral feeding. Nonetheless, patients’ tolerance may not be optimal and migration may occur, as well as other possible complications. The long-term efficacy and safety must be demonstrated by prospective trials.

#### ERCP Guidewires and Catheters As Adjunct Tools

In select cases, when the stenosis is so severe that the lumen cannot be identified and passed, guidewires used for can be used (111). After preloading, a standard ERCP catheter with a 0.035″ guidewire, the floppy tip can be used to gently probe the stricture. Once the lumen has been identified and passed, confirmation of the final guidewire tip location should be monitored with fluoroscopic guidance. Then, the guidewire may be left in place and used for dilations with Savary–Gilliard polyvinyl or ERCP dilators.

## Surgical Management

Although conservative treatment is preferable for ASs (39), children who fail to respond to all conservative strategies require a surgical intervention.

Despite the difficulties in the management of refractory and recurrent ASs, the number of reported patients who require resection of the stricture is remarkably small, ranging from 3 to 7% (12, 112). No large data are available about long-term outcomes of these patients, since cohorts are small and data are always retrospective.

### Stricture Resection with Direct Anastomosis

Resection and esophageal anastomosis is the most common surgical intervention for refractory ASs (112, 113). Although mediastinal scarring complicates reoperation, by this time the esophageal ends are in apposition and better vascularized, increasing the likelihood of success (114). Nonetheless, patients treated with a second end-to-end anastomosis may still require postoperative dilatation, as well as a second operative revision (12).

### Esophageal Replacement

Interposition graft placement for the treatment of AS (as opposed to the primary treatment of long-gap EA) is exceedingly rare in the recent literature (12).

The decision to abandon the native esophagus and perform replacement surgery is an important one and needs to be a well-informed decision, made by experienced surgeons in discussion with a multidisciplinary team (114). The morbidity associated in the long and short term with esophageal replacement may be significant, but the benefits are also easily seen in those patients with a long and complicated previous surgical history (114).

The choice of graft (gastric transposition, colon interposition, jejunal interposition, and gastric tube) is determined by individual and institutional expertise. Outcomes of the different approaches must be prospectively evaluated. A recent review aimed to describe *pros and cons* of each technique, regardless of the indication (115).

## Conclusion

With better survival from improved surgical techniques and preoperative/postoperative care, we are increasingly seeing children with EA experiencing significant short- and long-term gastrointestinal morbidities. Despite the real incidence is still undetermined, AS is the most common complication following EA operative repair. Despite the efforts to identify possible pre-, intra-, and postoperative risk factors, incidence of ASs does not seem to be changed over time. Limited evidence exists regarding diagnosis and treatment, and there is still a lack of uniform and systematic approach for the care of these patients. By virtue of this, over the last year, the working group of International Network on Esophageal Atresia, including members from ESPGHAN and NASPGHAN, published the first evidence-based Guidelines for the management of children with EA.

It is strongly recommended that ASs are investigated and treated only when symptoms occur as opposed to routine screening and dilatation. The diagnosis can be done by either contrast study or endoscopically. The mainstay of stricture management is serial esophageal dilatation. No clear advantage of either balloon or bougie dilator has been demonstrated; therefore, dilation should be carried out using the technique with which the operator is most skilled and experienced. Despite several dilation sessions, AS persistency or recurrence may be experienced. In these cases, conservative approach should be preferred before considering any surgical treatment. Different non-surgical adjuvant treatments have been used to minimize the risk of stricture reoccurrence. Although data are scarce and heterogeneous, especially in EA patients, temporary stent placement or application of topical MMC following dilation are suggested as a first-line adjunctive therapy.

**Figure 1:**
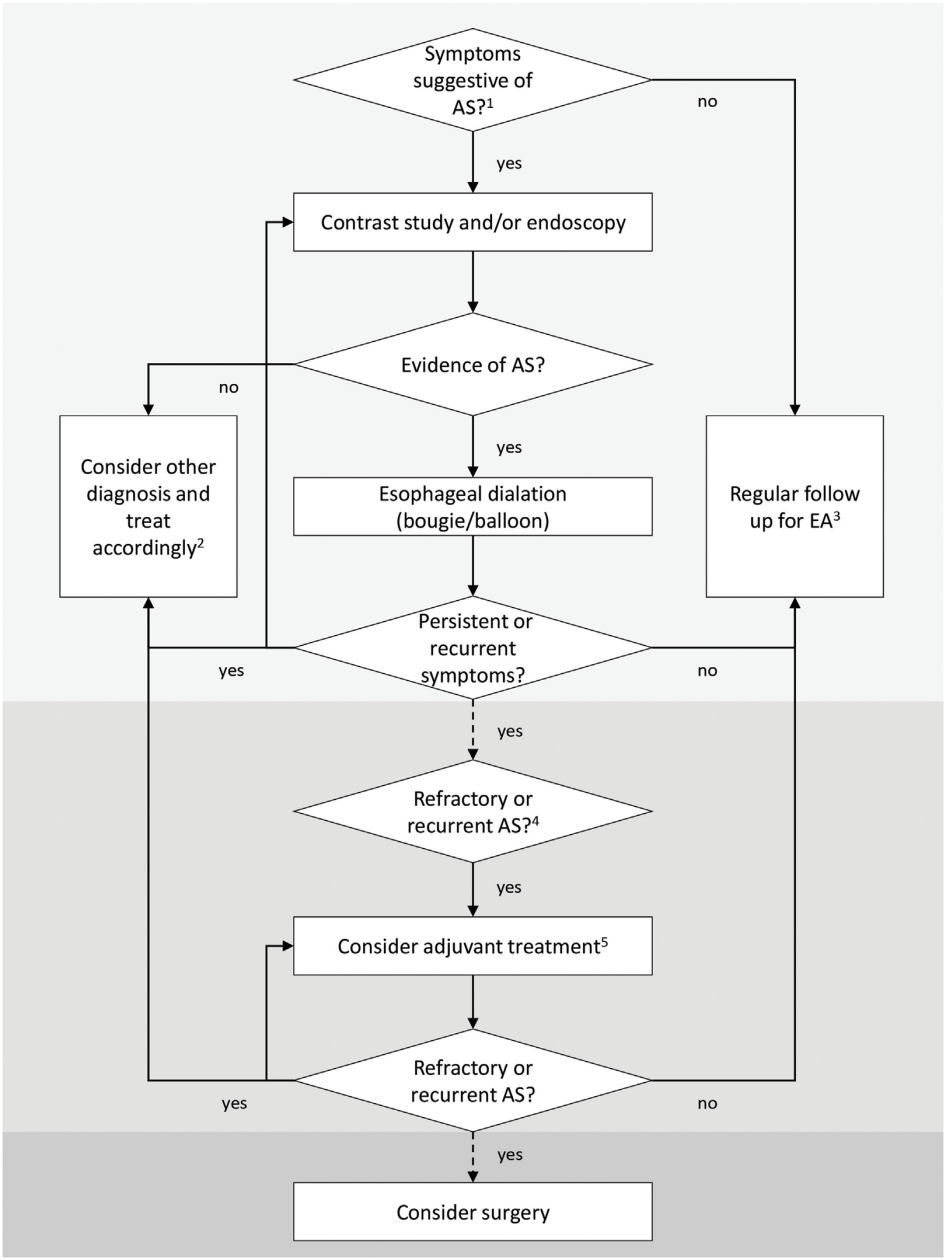
**Simplified algorithm for diagnosis and treatment of anastomotic strictures (ASs) after esophageal atresia (EA) repair**. ^1^Symptoms suggestive of AS depend upon the age of the child and the type of food ingested (liquid or solid) and include feeding and swallowing difficulties, regurgitation and vomiting, mucus or food impaction, cough, drooling, recurrent respiratory infections, foreign body impaction, and poor weight gain. In EA patients, these symptoms may overlap with other pathologic conditions, and none of them alone is sensitive or specific enough to diagnose an AS (14). ^2^Other diagnosis includes esophageal dysmotility, recurrent tracheoesophageal fistula, gastroesophageal reflux disease, tracheomalacia, laryngeal clefts, and vocal cord dysfunction; these conditions may coexist and exacerbate AS symptoms. Patients with EA should be evaluated regularly by a multidisciplinary team (14). ^3^EA children in the first 2 years of life (with special attention during the introduction of solid food) and patients with long-gap EA and postoperative anastomotic leak need a closer follow-up (14). ^4^Recurrent AS: ≥3 episodes of clinically relevant stricture relapses after dilations (14) or inability to maintain a satisfactory luminal diameter for 4 weeks once the age-appropriate feeding diameter has been achieved (20). Refractory AS: inability to successfully remediate the anatomic problem to obtain age-appropriate feeding possibilities after a maximum of five dilation sessions (refractory) with maximal 4-week intervals (20). ^5^Potential adjuvant treatments may include intralesional and/or systemic steroids, topical application of mitomycin C (MMC), stents, and an endoscopic incisional therapy (14). Temporary stent placement or application of topical MMC following dilation is suggested as a first-line adjunctive treatment in children (20).

Even though recent Guidelines from ESPGHAN and NASPGHAN have provided an essential help for the management of EA patients in clinical practice, there is still an overall lack of evidence-based indications and several questions have no answers yet. Large, prospective, multicenter studies are needed to better understand AS pathophysiology and to determine the optimal treatment strategy, especially in patients with refractory and recurrent AS. A simplified algorithm for diagnosis and treatment of AS in EA patients shown in Figure 1 is based on current knowledge.

## Author Contributions

RT and GA performed the literature search, analyzed the data, and drafted the manuscript. LD and PA designed and coordinated the work and critically revised the manuscript for important intellectual content. FT, TC, VB, AC, ER, FR, SF, and GFA contributed to literature search and data interpretation and critically revised the manuscript. All the authors approved the final version of the manuscript.

## Conflict of Interest Statement

The authors declare that the research was conducted in the absence of any commercial or financial relationships that could be construed as a potential conflict of interest. The reviewer, MH-L, and the handling editor declared their shared affiliation, and the handling editor states that the process nevertheless met the standards of a fair and objective review.
